# Food-evoked nostalgia as a mediator between food-related satisfaction and psychological distress among older adults: a structural equation modeling approach

**DOI:** 10.3389/fpubh.2026.1776433

**Published:** 2026-04-29

**Authors:** Amani Alhazmi, Manal Mohammed Hawash, Mona Metwally El-Sayed, Mahmoud Abdelwahab Khedr, Zainab Eid Kmal Klila, Eman Mahmoud Mohammed Shoukr

**Affiliations:** 1 Public Health Department, Applied Medical Sciences College, King Khalid University, Abha, Saudi Arabia; 2 Department of Community, Psychiatric, and Mental Health Nursing, College of Nursing, Qassim University, Buraydah, Saudi Arabia; 3 Psychiatric and Mental Health Nursing Department, Alexandria University, Alexandria, Egypt; 4 Department of Psychiatric and Mental Health Nursing, Faculty of Nursing, Port-Said University, Port-Said, Egypt; 5 Gerontological Nursing Department, Faculty of Nursing, Alexandria University, Alexandria, Egypt; 6 Community Health Nursing Department, College of Nursing, Jouf University, Sakaka, Saudi Arabia

**Keywords:** emotional wellbeing, food satisfaction, food-evoked nostalgia, older adults, psychological distress, Saudi Arabia

## Abstract

**Background:**

Food-evoked nostalgia affects emotional experiences and mental health, but its impact on older adults, a group for whom food memories have strong personal significance, is still not well understood.

**Objective:**

This study examined the mediating role of food-evoked nostalgia in the relationship between food satisfaction and psychological distress (depression, anxiety, and stress) among older adults.

**Methods:**

A cross-sectional study was conducted among 300 community-dwelling older adults (≥60 years) attending five primary healthcare centers in Abha City, Saudi Arabia. Data were collected through structured face-to-face interviews using validated Arabic versions of the Satisfaction with Food-Related Life (SWFL), State Functions of Nostalgia Scale (SFNS), and Depression Anxiety Stress Scale-21 (DASS-21). Statistical analyses included descriptive statistics, Pearson correlations, and the latent variable structural equation model (SEM) using SPSS and AMOS (version 26). Common method bias, discriminant validity, and multicollinearity were assessed using Harman’s single-factor test, confirmatory factor analysis, and variance inflation factors, respectively.

**Results:**

Participants reported high levels of nostalgia (*M* = 88.3, SD = 24.8) and food satisfaction (*M* = 16.2, SD = 4.0). Food-evoked nostalgia was positively correlated with food satisfaction (*r* = 0.97, *p* < 0.001) and negatively correlated with depression, anxiety, and stress (*r* ≈ −0.95, *p* < 0.001). The latent variable structural equation model indicated that food-evoked nostalgia was a significant partial mediator in the relationship between food satisfaction and emotional status. Food satisfaction was strongly positively associated with food-evoked nostalgia (*β* = 0.97), which in turn was negatively associated with depression (*β* = −0.64), anxiety (*β* = −0.63), and stress (*β* = −0.67). Food satisfaction also showed direct negative associations with depression (*β* = −0.32), anxiety (*β* = −0.33), and stress (*β* = −0.29). All paths were statistically significant (*p* < 0.001). Incremental fit indices (*χ*^2^/df = 2.84, CFI = 0.94, IFI = 0.94, NFI = 0.92, RMSEA = 0.052; 90% CI: 0.048–0.056) indicated a good model fit.

**Conclusion:**

Food-evoked nostalgia is associated with lower levels of psychological distress and may function as a psychological resource linking food satisfaction to emotional outcomes. Incorporating culturally meaningful food experiences may help support mental health among older adults by reinforcing emotional connections, fostering positive nostalgic memories, and contributing to improved emotional wellbeing.

## 1 Introduction

Food plays a crucial role throughout the human lifespan, extending beyond mere sustenance to encompass cultural, emotional, and psychosocial contexts. Exploring the intrinsic relationship between eating experiences and wellbeing is crucial for the preservation of regional food culture and the enhancement of cultural identity (1). Not only does food produce physiological experiences such as lethargy, hunger, illness, and discomfort, but it also influences psychological experiences, both of which can shape a person’s sense of wellbeing. Through eating, sensory pleasure, familiarity, and emotional resonance can act as an initial spark for satisfaction (2, 3).

Food-related satisfaction refers to the degree to which individuals feel content with their dietary experiences, including taste, variety, and cultural relevance (4, 5). Food satisfaction is linked to both physical health and psychological outcomes, as dietary habits foster positive emotions, reduce stress, and reinforce social bonds, ultimately leading to overall life satisfaction (2, 5–7). For older adults, food often carries heightened significance, as it evokes autobiographical memories, reinforces cultural belonging, and provides comfort during the challenges of aging (5). Nakabayashi et al. (8) also demonstrated that traditional food consumption is a significant predictor of satisfaction with food-related life. In Saudi society, where communal dining and traditional dishes hold deep cultural and social significance, food-related satisfaction may have a particularly profound impact on overall wellbeing. Nevertheless, aging often brings about physiological and psychosocial challenges that can diminish food-related satisfaction and increase vulnerability to adverse emotional outcomes (7).

Saudi Arabia is undergoing rapid demographic and social changes, characterized by an increasing aging population alongside changes in traditional family structures. This shift highlights the vulnerability of older adults to reduced familial support and social isolation, underscoring the need to address the emotional health of older adults (9). As people age, they often face loneliness, anxiety, social isolation, and depression, which usually result from losses in health, loved ones, purpose, or autonomy. They also frequently experience age-related declines in appetite, sensory functions, oral health, and dietary restrictions (10, 11). Such difficulties are exacerbated by the prevalence of aging-related chronic conditions and multimorbidity, both of which further compromise the quality of life among Saudi older adults (9). On the other hand, aging also brings resilience, emotional regulation, and wisdom. Older adults often prioritize meaningful relationships and show greater acceptance and calmness, traits that are associated with superior emotional wellbeing compared to younger cohorts. Hence, understanding how older adults maintain emotional health despite these challenges requires examining their cultural and psychological mechanisms, such as nostalgia, which is considered an important factor in promoting health and wellbeing in aging populations (12, 13).

Nostalgia is a bittersweet, wistful feeling and a sentimental longing for the past, which is universally experienced across cultures and throughout life (13). Food-evoked nostalgia occurs when specific flavors or meals trigger memories of meaningful past experiences, often associated with family, cultural traditions, or significant life events (6). Reid et al. (14) found that food uniquely triggers nostalgia, reaching beyond sensory gratification to foster a deeper emotional resonance, predominantly positive emotional experiences, which are linked to key psychological outcomes, including positive affect, self-esteem, social connection, and a sense of meaning in life. This is supported by the Broaden-and-Build Theory, which posits that positive emotions, such as those experienced during nostalgia, broaden individuals’ thought–action repertoires and build lasting psychological resources, thereby strengthening resilience and reducing psychological distress (15, 16). Research suggests that nostalgia serves as a psychological resource to buffer against psychological distress, enhancing mood, fostering a sense of continuity, and mitigating feelings of loneliness, thereby helping individuals manage stress, anxiety, and depression (5, 17).

For aging individuals, nostalgia can be particularly potent, as food memories often span decades and are deeply tied to personal and cultural identities. It evokes deep-seated memories and emotions, contributing significantly to their overall wellbeing (5, 18). This aligns with the Continuity Theory of Aging, which posits that wellbeing is maintained by preserving familiar habits and self-concepts. According to this theory, older adults strive to preserve their internal structures, such as personality, values, and beliefs, as well as external structures, including routines, social roles, and relationships, by adapting strategies rooted in past experiences (19). Food-evoked nostalgia (FEN) connects present experiences with culturally meaningful traditions, ensuring identity stability and emotional continuity through deep-rooted sensory and cultural anchors (20). Beyond eliciting private reminiscences, food-evoked nostalgia can foster emotional comfort for older adults through social pathways (14). Simpson et al. (13) found that nostalgic food experiences foster social alignment, a valuable asset for older populations, particularly those facing isolation or loss, as they can serve as an emotional bridge to reinforce identity continuity and a sense of belonging throughout later life.

Despite the profound cultural role of food, which is closely tied to tradition, religious celebrations, family, and social interactions, ongoing modernization and globalization are shifting dietary habits toward Western foods. This transition may erode the cultural and emotional significance of traditional dishes, particularly those linked to religious celebrations and childhood memories within Saudi society (21). Positioning nostalgia as a mediator, therefore, provides a crucial lens for understanding how food-related satisfaction relates to psychological distress, offering a foundation for developing culturally sensitive interventions to support the mental health of Saudi Arabia’s older adult population.

Although scholarly attention to the psychology of food has grown, research examining the combined influence of food-related satisfaction and nostalgia on psychological distress remains limited in the MENA region. Research in this area could inform the development of targeted strategies that harness the therapeutic potential of nostalgia and food to promote health and enhance emotional wellbeing, particularly among older adults. In Saudi Arabia, exploring these dynamics not only broadens cross-cultural perspectives but also highlights how traditional food practices can serve as low-cost, culturally grounded interventions to support emotional wellbeing in later life. Accordingly, this study aimed to examine the mediating role of food-evoked nostalgia in the relationship between food-related satisfaction and psychological distress among older adults in Saudi Arabia, addressing gaps in the literature on how cultural and emotional dimensions of food influence mental health in aging populations. The study also tests the following hypotheses: (H1) greater food-related satisfaction is associated with higher levels of food-evoked nostalgia and lower levels of stress, anxiety, and depression among Saudi older adults; (H2) there is a significant positive relationship between food-related satisfaction and food-evoked nostalgia; (H3) there is a significant positive relationship between food-evoked nostalgia and psychological distress; and (H4) food-evoked nostalgia mediates the relationship between food-related satisfaction and psychological distress among Saudi older adults.

## 2 Materials and methods

### 2.1 Study design

This study employed a descriptive cross-sectional design that adheres to the Strengthening the Reporting of Observational Studies in Epidemiology (STROBE) checklist guidelines (22).

### 2.2 Study setting and population

The study was conducted in primary health care centers (PHCCs) located in the Abha district of the Asir region, Saudi Arabia. These PHCCs operate under the Ministry of Health and provide comprehensive services to individuals across all age groups, including preventive care, diagnosis, treatment, and rehabilitation for a wide range of health conditions. The study population comprised community-dwelling older adults who regularly attended these PHCCs. Eligibility criteria included being 60 years of age or older, demonstrating adequate cognitive function, and providing informed consent to participate. Additional inclusion requirements specified that participants must have a stable living situation, be able to prepare meals independently or with minimal assistance, and be familiar with traditional Saudi foods. Exclusion criteria encompassed individuals exhibiting severe clinical depression (PHQ-9 score ≥20) and those with significant cognitive impairment, defined by a Mini-Mental State Examination-Second Edition (MMSE-2) score of 20 or lower.

### 2.3 Sample size calculation

Using G*Power version 3.1.9.7A, we estimated the required sample size with power (1–*β*) = 0.95, effect size = 0.15, and *α* = 0.05, for one group and one measurement based on prior studies (23, 24). The calculation yielded 250 participants. To accommodate potential nonresponse and dropout, a 20% buffer (25) was added, resulting in a final sample of 300 older adults.

### 2.4 Sampling and recruitment process

A multistage probability sampling technique was employed to recruit participants for this study. In the first stage, a comprehensive list of the 10 primary healthcare centers (PHCCs) in Abha City was obtained from the Ministry of Health, which served as the initial sampling frame. From this list, five PHCCs were randomly selected using Python’s random sampling function without replacement to ensure unbiased selection. Following institutional approval, permission was obtained from the principal of each selected PHCC to facilitate participant recruitment. In the second stage, each participating PHCC provided a list of attending older adults, which served as the secondary sampling frame. Using the Research Randomizer (version 0.4) tool, a predetermined number of participants were randomly selected from each list. To ensure proportional representation across centers, the sample size for each PHCC was allocated based on its average weekly patient visit frequency, which served as a proxy measure for the size of the older adult population attending the center. Trained researchers collected data through structured, face-to-face interviews conducted in a private setting within each PHCC. The survey first invited approximately 321 older adults to participate. However, seven participants with depressive symptoms and 9 with cognitive impairment were excluded, and 5 participants refused to participate. Thus, the final sample comprises 300 older adults, with a response rate of 98% and a dropout rate of 2%.

### 2.5 Instruments of data collection

Data for this study were collected using the Older Adults’ Personal Characteristics Data Sheet, the State Functions of Nostalgia Scale (SFNS), the Satisfaction with Food-Related Life (SWFL) Scale, and the Depression Anxiety Stress Scale-21 (DASS-21). Additionally, two other instruments were used to exclude participants with depression or cognitive deficits, thereby maintaining homogeneity and group consistency: the Patient Health Questionnaire-9 (PHQ-9) and the Mini-Mental State Examination, Second Edition (MMSE-2). Since the survey was conducted in Arabic, a back-translation procedure was implemented to ensure semantic consistency, given that the original measures were developed in English.

#### 2.5.1 Older adults’ personal characteristics data sheet

The data sheet includes participants’ age, gender, marital status, educational level, occupation, and financial status. A questionnaire assessing lifestyle patterns was also administered, covering diet, sleep, smoking habits, and physical exercise. Each participant’s height and weight were measured three times using a portable electronic scale (Omron, BF508, Japan), with weights recorded to the nearest 0.1 kg. Height was measured with a tape measure against a straight wall, to the nearest 0.5 cm. Body Mass Index (BMI) was calculated for each participant by dividing their weight in kilograms by the square of their height in meters. Weight status was classified according to WHO criteria, categorizing participants as underweight (<18.5), average weight (18.5–24.9), overweight (25.0–29.9), or obese (≥30) (26).

#### 2.5.2 Patient Health Questionnaire-9 (PHQ-9)

The PHQ-9 is a widely validated tool used to screen, diagnose, and measure the severity of depression. This self-administered nine-item questionnaire is based on the DSM-5 criteria for major depressive disorder (27). Each item is scored on a 4-point Likert scale, yielding a total score ranging from 0 to 27. Scores are categorized to reflect depression severity, from minimal (0–4) to severe (19–26), providing actionable insights for treatment planning and monitoring. In a study conducted by AlHadi et al. (28) in Saudi Arabia, the Arabic PHQ-9 demonstrated a Cronbach’s alpha of 0.857, indicating high internal consistency, demonstrating its effectiveness in assessing depression severity and treatment response. In this study, the PHQ-9 was administered solely during the eligibility screening phase to identify and exclude individuals with severe depressive symptoms (score ≥20).

#### 2.5.3 Mini-Mental State Examination, Second Edition (MMSE-2)

The MMSE-2 is among the most widely used cognitive screening tools globally, initially developed by Folstein and McHugh (29). The MMSE-2 typically requires 7–10 min to administer, assessing a range of cognitive domains, including orientation, recall, attention, calculation, language, and visuospatial skills. The MMSE-2 was evaluated against the Diagnostic and Statistical Manual of Mental Disorders, Fourth Edition, Text Revision (DSM-IV-TR) criteria for dementia. The test has a maximum score of 30, with performance stratified as follows: 27–30 is considered normal, 21–26 indicates mild cognitive impairment, 11–20 suggests moderate impairment, and ≤10 reflects severe impairment. The Arabic version of MMSE-2 has reported a Cronbach’s alpha of 0.72, indicating acceptable internal consistency. The test–retest reliability was high, with a correlation coefficient of 0.95 (30). In this study, participants with MMSE-2 scores of 20 or lower were excluded to ensure adequate cognitive function for reliable self-reporting.

#### 2.5.4 The State Functions of Nostalgia Scale (SFNS)

The State Functions of Nostalgia Scale (SFNS) was developed by Sedikides et al. (17). It is a 20-item instrument designed to assess the immediate, state-level psychological functions of nostalgia. Items are rated on a 7-point Likert scale from 1 (strongly disagree) to 7 (strongly agree) and are organized into four theoretically grounded subscales: Self-Positivity, Existential Meaning, Social Connectedness, and Positive Affect. Each subscale comprises five items, with scores computed by summing or averaging item responses; higher scores indicate greater endorsement of the respective nostalgic function. A total nostalgia score may also be derived from all 20 items. The original scale demonstrates sound psychometric properties, with confirmatory factor analysis supporting the four-factor structure and subscale Cronbach’s alpha values typically ranging from 0.83 to 0.90. In the present study, the SFNS was translated into Arabic using a back-translation procedure. To ensure conceptual and cultural appropriateness, the Arabic version was pilot-tested and demonstrated excellent internal consistency (*α* = 0.89). To examine the measurement properties of the nostalgia construct, confirmatory factor analysis (CFA) was conducted, and discriminant validity was evaluated using the Fornell–Larcker criterion (31). The analysis confirmed adequate discriminant validity between food-evoked nostalgia and related constructs (food satisfaction and depression), with the square root of the average variance extracted (AVE) for nostalgia (0.82) exceeding its inter-construct correlations (e.g., *r* = 0.71 with food satisfaction). Additionally, multicollinearity diagnostics revealed no significant concerns, with a variance inflation factor (VIF) of 2.14 for food-evoked nostalgia in the subsequent path analysis.

#### 2.5.5 Satisfaction with Food-Related Life (SWFL) Scale

This scale is a psychometric instrument developed by Grunert et al. (32) to assess individuals’ cognitive and affective evaluation of their overall food-related experiences. This concise five-item scale measures overall satisfaction with aspects of food in life, including meal enjoyment, food availability, and dietary contentment. Participants respond using a 5-point Likert scale (1 = strongly disagree to 5 = strongly agree), with higher scores indicating greater food-related life satisfaction, ranging from 5 to 25. The SWFL has shown strong psychometric properties across diverse populations. The scale demonstrated high internal consistency, with Cronbach’s alpha values ranging from 0.80 to 0.90 (33). After being translated into Arabic, all items were pre-tested. The scale maintained high validity, with factor loadings ranging from 0.58 to 0.89, which improved to 0.70 to 0.94 after varimax rotation, accounting for 78.35% of the variance. The Kaiser–Meyer–Olkin (KMO) measure was excellent at 0.950, and Bartlett’s test of sphericity was significant (*p* ≤ 0.001), confirming the data’s suitability for factor analysis. This Arabic version of the SWFL demonstrated good reliability (*α* = 0.82) in the current study.

#### 2.5.6 Depression Anxiety Stress Scale-21 (DASS-21)

This scale, developed by Lovibond and Lovibond (34), is a brief self-report instrument designed to assess emotional states of depression, anxiety, and stress. It comprises 21 items, divided into three subscales, each with seven items. Respondents rate how much each statement applied to them over the past week using a 4-point Likert scale ranging from 0 (Did not apply to me at all) to 3 (Applied to me very much or most of the time). Higher scores indicate greater severity of negative emotional states. Each subscale score ranges from 0 to 42, and the total score from 0 to 126, with higher scores reflecting greater emotional distress. Cutoff points categorize severity levels as follows: depression normal (0–9), mild (9–12), moderate (13–19), severe (20–26), extremely severe (28+); anxiety normal (0–7), mild (8, 35), moderate (9–13), severe (14–18), extremely severe (20+); and stress normal (0–14), mild (14–17), moderate (18–24), severe (25–32), extremely severe (34+). The original DASS-21 demonstrated excellent internal consistency, with Cronbach’s alpha values of 0.91 for depression, 0.84 for anxiety, 0.90 for stress, and 0.93 for the overall scale (34). The Arabic version of the DASS-21, which was translated and validated by Ali et al. (36) and used in this study, showed strong psychometric properties, with confirmatory factor analysis supporting the original three-factor structure and Cronbach’s alpha coefficients indicating high internal consistency (*α* = 0.83 for depression, 0.80 for anxiety, 0.82 for stress, and 0.84 for the total scale). The Arabic translation of the DASS-21 demonstrated excellent reliability in this study, with Cronbach’s alpha values of 0.94 for depression, 0.92 for anxiety, 0.90 for stress, and 0.91 for the total scale, confirming its suitability and internal consistency for assessing emotional distress among Arabic-speaking older adults.

### 2.6 Pilot study

Before the primary data collection phase, a pilot study was conducted to evaluate the clarity, comprehensibility, and cultural appropriateness of the Arabic translations of the instruments, as well as the feasibility of the overall data collection process. The pilot was conducted on 30 older adults recruited from a PHCC not included in the main study sample to prevent contamination. Participants in the pilot study were drawn from the same target demographic as the main study population. They completed the full set of instruments under conditions identical to those planned for the main survey. Feedback from participants was solicited regarding the clarity of wording, length of the interview, and any items perceived as ambiguous or culturally sensitive. Minor linguistic adjustments were made to improve the readability and contextual relevance of certain items without altering the original meaning. The pilot data were also used to test the data entry template and the logistical workflow between PHCCs and the research team. No major procedural issues were identified, indicating the feasibility and clarity of the study tools and methods. The pilot data were excluded from the final statistical analyses.

### 2.7 Validity and reliability

The study employed rigorous methods to establish the validity and reliability of the measurement instruments. For content validity, a panel of five experts in geriatric nursing, nutrition, and public health evaluated the cultural appropriateness and relevance of the Arabic translations of the SFNS and SWFL scales. Their feedback led to minor wording changes to better align with Saudi cultural norms, while preserving the original constructs. Construct validity was examined via factor analysis: SFNS supported the expected four-factor structure, and SWFL supported a unidimensional structure. Sampling adequacy was excellent (KMO > 0.95), and Bartlett’s test was significant (*p* < 0.001). Reliability was evaluated through internal consistency. Cronbach’s alpha coefficients demonstrated excellent reliability for all scales: SFNS (*α* = 0.89), SWFL (*α* = 0.82), and DASS-21(*α* = 0.91).

### 2.8 Data collection procedures

Data were collected between July and September 2025 at five selected PHCCs in Abha City. Before starting data collection, the research team obtained official approval from each PHCC’s administration to facilitate participant access and coordination. A team of trained researchers, each with a background in nursing or public health, administered the instruments. Data were gathered through structured, face-to-face interviews conducted in Arabic within private consultation rooms at each PHCC to ensure participants’ comfort and privacy. Each interview lasted about 20 to 30 min. Participants were first screened using the Patient Health Questionnaire-9 (PHQ-9) and the Mini-Mental State Examination, Second Edition (MMSE-2) examination to confirm eligibility. Before the interviews, researchers explained the study’s purpose and obtained written informed consent from each participant. Participants were reassured that their involvement was voluntary and that they could withdraw at any time without consequences. Before completing the main instruments, participants were guided through a standardized food-related nostalgia induction to elicit memories of their food experiences. During this induction, the interviewer prompted participants to recall and mentally visualize a past event centered around a meal, dish, or food experience that elicited nostalgic emotions, particularly those connected to culturally familiar Saudi foods. Participants were given approximately 5 min to visualize the memory before completing all scales. This induction was specifically designed to anchor participants’ responses on SFNS to food-related experiences within the Saudi cultural context, while preserving the original structure and psychometric properties of the scale. This procedure also functioned as a cognitive priming step, ensuring that participants’ nostalgic state at the time of responding to the SFNS was temporally and contextually anchored to food-related memories rather than to general autobiographical nostalgia. The use of mood induction to activate state nostalgia is well-established in the literature (14, 17, 37). Immediately following the nostalgia induction, the SFNS was administered. Identical instructions and timing were used for all participants to ensure procedural consistency. The questionnaires were primarily self-administered; however, for participants with limited literacy, the interviewer read each item aloud verbatim in a neutral tone to minimize interviewer bias. Responses were recorded directly on printed forms or electronic tablets, depending on the resources available at each PHCC. At the end of each day, all completed questionnaires were reviewed and verified for accuracy, completeness, and internal consistency. The data were then securely stored in locked cabinets and password-protected digital folders accessible only to the principal investigator and authorized researchers.

### 2.9 Ethical consideration

The study was conducted in accordance with the Declaration of Helsinki and was approved by the Research Ethics Committee at King Khalid University under Approval No. KKU-53-2025-17. Written informed consent was obtained from all participants who chose to participate in the study. All participants were cognitively intact (MMSE-2 score > 20) and provided consent themselves; no proxy consent from legal guardians or next of kin was required or obtained. The questionnaires ensured anonymity and data protection, with access restricted to the research team.

### 2.10 Statistical analyses

Data were analyzed using IBM SPSS Statistics and SPSS AMOS version 26 (38). Descriptive statistics (mean, standard deviation, frequency, and percentage) were used to summarize socio-demographic, clinical, and study variables, and internal consistency of all scales was assessed using Cronbach’s alpha. Pearson’s correlation coefficients were used to examine the bivariate relationships among nostalgia, food satisfaction, and emotional distress (depression, anxiety, and stress). To assess potential common method bias, Harman’s single-factor test was conducted (39). The unrotated factor solution revealed that a single factor accounted for 32.4% of the total variance, below the recommended threshold of 50%, indicating that common method bias was not a significant concern. Prior to testing the structural relationships, confirmatory factor analysis (CFA) was conducted to evaluate the measurement properties of all constructs (31). The measurement model included food-evoked nostalgia (modeled as a second-order factor with four first-order dimensions), food satisfaction (a single factor), and depression, anxiety, and stress (each as a single factor). The measurement model demonstrated acceptable fit: *χ*^2^/df = 1.73, CFI = 0.94, IFI = 0.94, NFI = 0.92, RMSEA = 0.049, indicating adequate convergent validity with all factor loadings statistically significant (*p* < 0.001) (40, 41). Discriminant validity was evaluated using the Fornell–Larcker criterion (31), where the square root of the average variance extracted (√AVE) for each construct exceeded its inter-construct correlations, confirming empirical distinctness. Multicollinearity among predictor variables was assessed using the variance inflation factor (VIF). VIF values were 2.14 for nostalgia and 1.98 for food satisfaction, both below the conservative threshold of 3.0, indicating no multicollinearity concerns (40). Following confirmation of adequate measurement properties, a latent variable structural equation model (SEM) was estimated using maximum likelihood estimation to test the hypothesized mediation relationships (38, 41, 42). Model fit was assessed using multiple goodness-of-fit indices, including the chi-square (*χ*^2^), comparative fit index (CFI), incremental fit index (IFI), normed fit index (NFI), and root mean square error of approximation (RMSEA) (43, 44). The latent SEM demonstrated good fit to the data: *χ*^2^/df = 2.84, CFI = 0.94, IFI = 0.94, NFI = 0.92, RMSEA = 0.052 (90% CI: 0.048–0.056). The direct and indirect (mediated) effects were evaluated using standardized regression weights, standard errors (SEs), and critical ratios (CRs), with statistical significance set at *p* < 0.05 (two-tailed) (38, 41–43).

## 3 Results

Table 1 shows that more than half of the participants are aged 60–69 (54.0%), with 32.7% aged 70–79, and just 13.3% aged 80 or older, indicating that the sample is weighted toward younger older adults. Female participants predominate (61.0%) over males (39.0%). Most of the group (82.6%) have only a basic education, while secondary and university education levels are far less common (15.7 and 1.7%, respectively). In terms of marital status, 84.7% are married, 6.7% are widowed, 7.3% are divorced, and 1.3% are single. Regarding employment, 88.3% are not currently working, and 11.7% are employed. On income, 29.0% report it is not sufficient, 68.7% say it is somewhat sufficient, and only 2.3% consider it entirely sufficient. The place of residence is nearly evenly split, with 50.7% living in urban areas and 49.3% in rural areas. Most older adults live with their family (87.0%), while 8.0% live alone and 5.0% reside in assisted living facilities. Regarding meal patterns, 65.7% consume three meals daily, 27.0% have fewer than three meals, and 7.3% have more than three meals. Dependence on others for food preparation is reported by 46.7%, whereas 53.3% prepare their own meals. The majority (87.3%) eat with others, while 12.7% eat alone. Clinically, 96.0% report one or more chronic diseases, and 74.3% are taking medications.

**Table 1:** Socio-demographic and clinical characteristics of the study participants (*N* = 300).

Socio-demographic and clinical characteristics	Total (*N* = 300)	
	*n*	%
Age (years)		
60-	162	54.0
70-	98	32.7
80+	40	13.3
Gender		
Male	117	39.0
Female	183	61.0
Level of education		
Basic education	248	82.6
Secondary education	47	15.7
University and more	5	1.7
Marital status		
Single	4	1.3
Married	254	84.7
Widow	20	6.7
Divorced	22	7.3
Current work		
No	265	88.3
Yes	35	11.7
Income		
Not enough	87	29.0
Enough to some extent	206	68.7
enough	7	2.3
Residence		
Urban	152	50.7
Rural	148	49.3
Living arrangement		
Alone	24	8.0
With family	261	87.0
Assisted living facility	15	5.0
Number of meals		
Less than three meals	81	27.0
3 meals	197	65.7
More than three meals	22	7.3
Depending on others for food preparation		
No	160	53.3
Yes	140	46.7
Eating with others		
No	38	12.7
Yes	262	87.3
Chronic diseases		
No	12	4.0
Yes	288	96.0
Consuming medications		
No	77	25.7
Yes	223	74.3

Table 2 shows that a majority of older adults reported high levels of food-evoked nostalgia (62.3%), with a mean score of 88.30 (SD = 24.81). Similarly, over half of the participants (54.7%) reported high satisfaction with their food-related life, yielding a mean score of 16.22 (SD = 4.05). Regarding emotional wellbeing, as measured by the DASS-21, the findings reveal notable levels of psychological distress. For depression, the majority of participants fell into either the mild (46.3%) or moderate (46.3%) categories. For anxiety, an overwhelming majority (91.3%) were classified in the moderate range. In contrast, stress levels were predominantly normal (64.7%), although a substantial portion experienced moderate (29.7%) or severe (5.7%) stress. The mean scores for depression (*M* = 9.08, SD = 4.86), anxiety (*M* = 9.10, SD = 4.88), and stress (*M* = 9.06, SD = 4.85) were relatively similar.

**Table 2:** Descriptive statistics of the study variables (*N* = 300).

Variables categories	*n*	%	Min.–Max.	Mean (SD)
Food-evoked nostalgia				
Low	106	35.3	50–112	88.30 (24.807)
Moderate	7	2.3		
High	187	62.3		
Food-related satisfaction				
Low	79	26.3	8–21	16.22 (4.050)
Moderate	57	19.0		
High	164	54.7		
Depression				
Normal	22	7.4	2–18	9.08 (4.856)
Mild	139	46.3		
Moderate	139	46.3		
Severe	0	0.0		
Extremely severe	0	0.0		
Anxiety				
Normal	4	1.4	3–19	9.10 (4.875)
Mild	22	7.3		
Moderate	274	91.3		
Severe	0	0.0		
Extremely severe	0	0.0		
Stress				
Normal	194	64.7	3–18	9.06 (4.852)
Moderate	89	29.7		
Severe	17	5.7		
Extremely severe	0	0.0		

SD, standard deviation; Min.-Max., minimum and maximum scores; SFNS, State Functions of Nostalgia Scale (range: 20–140); SWFL, Satisfaction with Food-Related Life Scale (range: 5–25); DASS-21, Depression Anxiety Stress Scale-21. DASS-21: Depression: normal (0–9), mild (9–12), moderate (13–19), extremely severe (21+); anxiety: normal (0–7), mild (8, 35), moderate (9–13), extremely severe (15+); stress: normal (0–14), severe (14–17), extremely severe (19+).

Table 3 reveals that older adults’ feelings of nostalgia evoked by food were positively correlated with food satisfaction (r = 0.971, *p* < 0.001), indicating that as the nostalgic sentiment evoked by food increases, so does food satisfaction in this sample. Conversely, nostalgia is strongly and negatively associated with depression, anxiety, and stress (*r* ≈ −0.95 for each, *p* < 0.001), suggesting that higher nostalgia is associated with lower levels of these adverse emotional status. Food satisfaction likewise shows robust negative correlations with depression, anxiety, and stress (*r* values ≈ − 0.94, all *p* < 0.001). Among the emotional status variables themselves, depression, anxiety, and stress are all highly positively correlated with one another (e.g., depression anxiety *r* ≈ 0.944, anxiety–stress *r* ≈ 0.940, all *p* < 0.001).

**Table 3:** Correlation coefficients between study variables (*N* = 300).

Variables		Food-evoked nostalgia	Food satisfaction	Depression	Anxiety	Stress
Food-evoked nostalgia	*r*	1				
	*p*					
Food satisfaction	*r*	0.970^**^	1			
	*p*	<0.001				
Depression	*r*	−0.948^**^	−0.938^**^	1		
	*p*	<0.001	<0.001			
Anxiety	*r*	−0.952^**^	−0.943^**^	0.944^**^	1	
	*p*	<0.001	<0.001	<0.001		
Stress	*r*	−0.952^**^	−0.941^**^	0.944^**^	0.940**	1
	*p*	<0.001	<0.001	<0.001	<0.001	

*r*, Pearson correlation coefficients; ^**^p < 0.001 (two-tailed).

**Figure 1:**
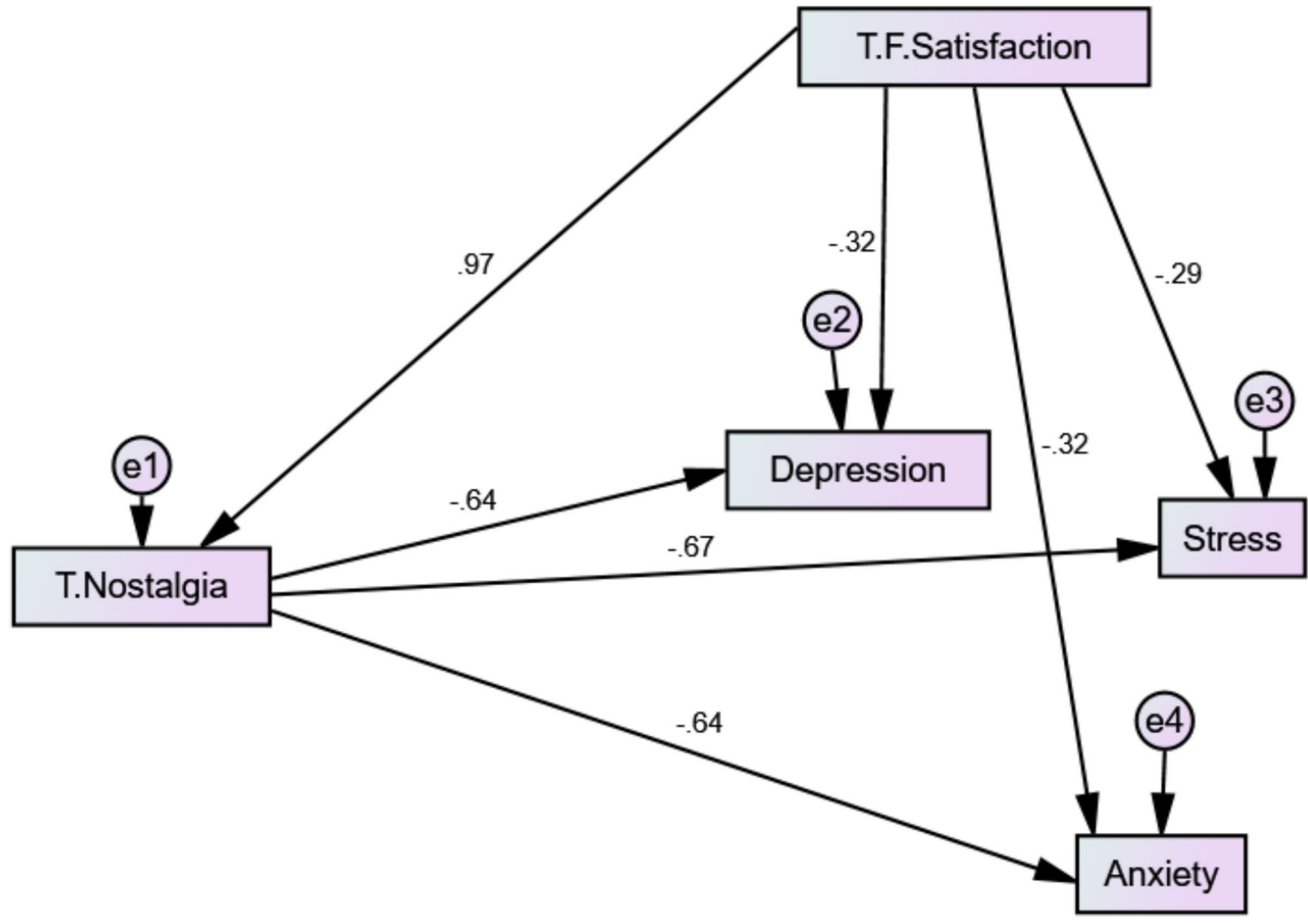
SEM showing the relationships between food-related satisfaction, food-evoked nostalgia, and psychological distress.

Table 4 and Figure 1 present the results of the structural equation model (SEM) examining the direct and indirect effects of food related satisfaction on psychological distress (depression, anxiety, and stress), with food-evoked nostalgia (FEN) as a mediating variable. The findings indicate that food satisfaction had a statistically significant positive effect on food-evoked nostalgia (*β* = 0.97, *B* = 0.58, SE = 0.08, CR = 7.25, *p* < 0.001), suggesting that higher levels of food satisfaction are associated with increased nostalgic experiences related to food. Food-evoked nostalgia, in turn, demonstrated significant negative effects on all three emotional outcomes. Specifically, FEN was negatively associated with depression (*β* = −0.64, *B* = −0.41, SE = 0.06, CR = −6.83, *p* < 0.001), anxiety (*β* = −0.63, *B* = −0.39, SE = 0.06, CR = −6.50, *p* < 0.001), and stress (*β* = −0.67, *B* = −0.43, SE = 0.06, CR = −7.17, *p* < 0.001), indicating that higher levels of food-evoked nostalgia are linked to lower levels of emotional distress. In addition to these indirect effects, food satisfaction also exerted significant direct negative effects on psychological status. Specifically, food satisfaction was negatively associated with depression (*β* = −0.32, *B* = −0.22, SE = 0.05, CR = −4.40, *p* < 0.001), anxiety (*β* = −0.33, *B* = −0.23, SE = 0.05, CR = −4.60, *p* < 0.001), and stress (*β* = −0.29, *B* = −0.20, SE = 0.05, CR = −4.00, *p* < 0.001). Taken together, these findings provide evidence of partial mediation, as food satisfaction influences psychological status both directly and indirectly through food-evoked nostalgia. All reported paths were statistically significant at *p* < 0.001. The model demonstrated a good fit to the data, as indicated by *χ*^2^/df = 2.84, CFI = 0.94, IFI = 0.94, and NFI = 0.92. Additionally, the RMSEA value of 0.052 (90% CI: 0.048–0.056) indicates a good level of approximation error, further supporting the adequacy of the model.

**Table 4:** SEM direct and indirect paths (*N* = 300).

Path	Direction	Predictors	Standardized *β*	Unstandardized *B*	S.E.	C.R.	*p*
FEN	←	FS	0.97	0.58	0.08	7.25	<0.001
Depression	←	FEN	−0.64	−0.41	0.06	−6.83	<0.001
Anxiety	←	FEN	−0.64	−0.39	0.06	−6.50	<0.001
Stress	←	FEN	−0.67	−0.43	0.06	−7.17	<0.001
Depression	←	FS	−0.32	−0.22	0.05	−4.40	<0.001
Anxiety	←	FS	−0.32	−0.23	0.05	−4.60	<0.001
Stress	←	FS	−0.29	−0.20	0.05	−4.00	<0.001

*B*, unstandardized regression coefficient; *β*, standardized regression coefficient; SE, standard error; CR, critical ratio. CR values greater than |1.96| indicate statistical significance at *p* < 0.05; SEM, structural equation model; FEN, food-evoked nostalgia (modeled as a second-order latent construct); food satisfaction = latent construct measured using the SWFL scale; positive *β* values indicate a positive association between variables, whereas negative *β* values indicate an inverse association; model fit indices: *χ*^2^/df = 2.84, CFI = 0.94, IFI = 0.94, NFI = 0.92, RMSEA, root mean square error of approximation = 0.052 (90% CI: 0.048–0.056), indicating good model fit; all paths were statistically significant at *p* < 0.001.

Top of Form

Bottom of Form

## 4 Discussion

Food significantly influences cultural identity and emotional experiences, especially among older adults, who often associate specific meals with treasured memories and pivotal life moments (5). In Saudi Arabia, where traditional dishes and communal dining play vital roles in social interactions, the interplay between food, nostalgia, and emotional wellbeing is particularly evident (8). This study examines the mediating role of food-evoked nostalgia in the relationship between food-related satisfaction and emotional distress states (depression, anxiety, and stress) among older adults in Saudi Arabia.

The current findings demonstrate a significant relationship between food-evoked nostalgia, food satisfaction, and emotional states of depression, anxiety, and stress in older adults. The high prevalence of high nostalgia levels among participants suggests that food-related memories play an important role in shaping their emotional experiences. Nostalgia, characterized by a sentimental longing for the past, has been shown to evoke positive emotions and foster a sense of continuity, especially in older individuals (45). This emotional connection may enhance feelings of safety and stability, which are crucial for mental health in aging populations (12).

The results also demonstrate that food satisfaction reflects a positive perception of dietary experiences among the older adults surveyed. This aligns with existing research emphasizing the role of food-related satisfaction in improving overall life satisfaction and emotional wellbeing (46). In Saudi culture, traditional meals often evoke fond memories and nurture a sense of belonging, contributing to emotional fulfillment (8). The data suggest that when older adults derive satisfaction from their food experiences, it is associated with heightened nostalgia, thereby reinforcing their emotional connections to their cultural heritage.

Despite the positive perceptions of food satisfaction, the significant levels of depression and anxiety symptoms among participants emphasize concerns regarding this demographic’s mental health. The prevalence of mental health issues in older adults is well-documented, with loneliness and social isolation being major contributors (47, 48). Aging-related challenges, such as declining physical health and the loss of loved ones, can exacerbate feelings of anxiety and depression (49). Addressing these mental health challenges is essential, as they can overshadow the positive impacts of nostalgia and food satisfaction, ultimately diminishing the overall quality of life (50).

Additionally, stress data reveals a complex emotional landscape in older adults. Although many participants report normal stress levels, a notable portion experiences severe to extremely severe stress, indicating that even with food-related nostalgia and food satisfaction, other stressors may significantly impact emotional wellbeing (13). Efforts to promote healthy eating must take these emotional dimensions into account, integrating strategies that enhance food-related satisfaction while also addressing mental health concerns. By acknowledging the multidimensional nature of emotional health in older adults, healthcare professionals and policymakers can create more effective, culturally relevant interventions that utilize nostalgia and food satisfaction to improve mental health outcomes (51).

The correlation coefficient further highlights the significant relationships between nostalgia, food satisfaction, and emotional states in older adults. The strong positive correlation between food-evoked nostalgia and food satisfaction indicates that nostalgic feelings are closely linked to satisfaction with food experiences. This connection emphasizes the emotional significance of food, especially for older individuals, as familiar tastes and aromas can trigger a cascade of positive memories (14).

The negative correlations between food-related nostalgia, food satisfaction, and psychological distress (depression, anxiety, and stress) highlight the potential of nostalgia and food to serve as buffers against negative emotions. Higher levels of both food-evoked nostalgia and food satisfaction are associated with lower levels of depression, anxiety, and stress, demonstrating that positive memories and satisfying food experiences contribute to emotional resilience (52). This aligns with findings that indicate nostalgia can elevate mood, foster continuity, and alleviate feelings of loneliness—critical factors for older adults facing various life challenges (53).

Additionally, the positive correlations among depression, anxiety, and stress indicate that these emotional statuses often coexist. This interconnectedness suggests that interventions targeting one emotional state may have a positive impact on others. The strong associations among these variables emphasize the need for a comprehensive approach to mental health, considering the interplay between different emotional dimensions. Understanding these relationships enables healthcare providers and policymakers to develop integrated strategies that support the emotional wellbeing of older adults, leveraging the positive impact of nostalgia and food satisfaction (51).

The latent variable structural equation model used in this study clarifies the complex relationships between food-evoked nostalgia, food satisfaction, and emotional states such as depression, anxiety, and stress levels among older adults. The model shows that food-related nostalgia is significantly and positively associated with food satisfaction, suggesting that individuals with positive food-related memories are more likely to enjoy satisfying dining experiences. This finding is consistent with the literature, which emphasizes the emotional and psychological importance of food in nurturing nostalgia and wellbeing, especially for older adults who may depend on food-related memories for comfort and cultural connection (14, 54).

Furthermore, the SEM food satisfaction is significantly associated with lower levels of negative emotional states such as depression, anxiety, and stress. The significant negative pathways from food satisfaction to these emotional outcomes suggest that when older adults are satisfied with their food experiences, they are less likely to experience adverse psychological effects. This protective effect illuminates the importance of food satisfaction in promoting mental health among aging populations. Previous studies have similarly shown that positive food experiences can enhance emotional resilience and overall life satisfaction, which is vital for older adults navigating the challenges of aging (5, 52).

The direct negative effects of food-evoked nostalgia on depression, anxiety, and stress further illustrate the multifaceted role food-related nostalgia plays in emotional health. The findings suggest that food-evoked nostalgia is not only associated with enhanced food satisfaction but also directly associated with reduced levels of psychological distress. This aligns with the theoretical model, which indicates that nostalgia acts as a psychological resource, assisting individuals in managing stress and enhancing emotional stability (55). The evidence for both direct and indirect effects underscores the complexity of these relationships, suggesting that food-evoked nostalgia can be a valuable asset in promoting wellbeing among older adults.

### 4.1 Study’s strengths and limitations

This study has several notable strengths. Theoretically, it employs a novel integrative framework linking food satisfaction, food-evoked nostalgia, and emotional health, positioning nostalgia as a psychological resource that supports resilience in later life. Grounded in Broaden-and-Build and Continuity theories, the study offers a meaningful theoretical contribution while addressing an important public health concern related to psychosocial determinants of mental health in aging populations. Methodologically, the use of validated psychometric instruments (SWFL, SFNS, and DASS-21) with Arabic translations enhances the reliability and validity of the measurements. The incorporation of face-to-face interviews, along with pre-screening of participants’ cognitive and mental states using standardized tools (MMSE-2 and PHQ-9), further strengthens the rigor and validity of the sample. Analytically, the use of latent variable SEM enables simultaneous estimation of direct and indirect effects while accounting for measurement error, providing deeper insight into the mechanisms linking food satisfaction to emotional distress. Culturally, this study is among the few exploring how traditional food culture influences mental health in older adults in Saudi Arabia, contributing to culturally specific aging research in the Middle East and globally.

Despite the study’s valuable insights, numerous limitations should be considered. First, the cross-sectional nature of the study precludes the ability to establish definitive causal relationships between the variables. Accordingly, longitudinal studies are needed to establish causality and explore how the dynamics of food-related nostalgia, food satisfaction, and emotional wellbeing evolve over time. Second, reliance on self-reported data may introduce bias, as participants may underreport negative feelings or overstate positive experiences. Although face-to-face interviews improved data quality, they may have increased social desirability bias, which could partially explain the high correlation coefficients observed among key constructs. However, Harman’s single-factor test indicated that common method bias was not a significant concern. Third, the strong correlations between food-evoked nostalgia, food satisfaction, and emotional distress raised concerns about multicollinearity and discriminant validity. These were formally examined using confirmatory factor analysis and the Fornell–Larcker criterion, which confirmed adequate discriminant validity. Variance inflation factor (VIF) values were below the conservative threshold of 3.0, indicating no multicollinearity concerns. Fourth, regarding sampling and generalizability, data were collected only from older adults attending healthcare centers in Abha City. The findings may lack generalizability to the broader Saudi Arabian older population, particularly those who do not seek healthcare services or reside in other regions. The sample was characterized by a high proportion of individuals with chronic diseases and low educational diversity, which may introduce sampling bias and limit external validity. Fifth, the nostalgia induction may have primed participants for nostalgic responses, inflating correlations with emotional outcomes. It might have increased the salience of nostalgia, resulting in stronger associations. Future research should compare induced with spontaneous, food-evoked nostalgia to evaluate priming effects. Lastly, the study focused exclusively on food-related nostalgia and did not assess other forms of nostalgia that may affect emotional wellbeing. Future research should examine specific sensory and contextual elements of food-evoked nostalgia, such as particular dishes, aromas, preparation methods, or social contexts, that most strongly influence emotional wellbeing through intervention-based or prospective designs, which could inform more targeted strategies for older adults.

## 5 Conclusion

This study highlights the crucial role of food-evoked nostalgia in shaping the connection between food satisfaction and psychological distress among older adults in Saudi Arabia. The findings suggest that nostalgic feelings associated with food are positively linked to individuals’ satisfaction with their eating experiences and are associated with lower levels of depression, anxiety, and stress. By illustrating the connections between food-related nostalgia, food satisfaction, and psychological outcomes, this research highlights the importance of culturally relevant approaches in supporting the mental health of older adults facing various life challenges. These findings have practical implications for targeted interventions. Leveraging food’s cultural significance and nostalgia may promote mental health in older adults. Incorporating traditional foods and communal dining can strengthen emotional resilience and support wellbeing. These strategies could be used in community geriatric care, adult day centers, and nutritional programs. The study adds to the literature that positions nostalgia as a valuable psychological resource for aging populations. Recognizing the link between food, memory, and emotion can help develop holistic, culturally sensitive approaches to enhance mental health in later life.

## Data Availability

The raw data supporting the conclusions of this article will be made available by the authors, without undue reservation.
